# Blinded by Beauty: Attractiveness Bias and Accurate Perceptions of Academic Performance

**DOI:** 10.1371/journal.pone.0148284

**Published:** 2016-02-17

**Authors:** Sean N. Talamas, Kenneth I. Mavor, David I. Perrett

**Affiliations:** School of Psychology and Neuroscience, University of St Andrews, St Andrews, United Kingdom; University of Lincoln, UNITED KINGDOM

## Abstract

Despite the old adage not to ‘judge a book by its cover’, facial cues often guide first impressions and these first impressions guide our decisions. Literature suggests there are valid facial cues that assist us in assessing someone’s health or intelligence, but such cues are overshadowed by an ‘attractiveness halo’ whereby desirable attributions are preferentially ascribed to attractive people. The impact of the attractiveness halo effect on perceptions of academic performance in the classroom is concerning as this has shown to influence students’ future performance. We investigated the limiting effects of the attractiveness halo on perceptions of actual academic performance in faces of 100 university students. Given the ambiguity and various perspectives on the definition of intelligence and the growing consensus on the importance of conscientiousness over intelligence in predicting actual academic performance, we also investigated whether perceived conscientiousness was a more accurate predictor of academic performance than perceived intelligence. Perceived conscientiousness was found to be a better predictor of actual academic performance when compared to perceived intelligence and perceived academic performance, and accuracy was improved when controlling for the influence of attractiveness on judgments. These findings emphasize the misleading effect of attractiveness on the accuracy of first impressions of competence, which can have serious consequences in areas such as education and hiring. The findings also have implications for future research investigating impression accuracy based on facial stimuli.

## Introduction

A review by Langlois et al. [1] suggested that people regularly make judgements based on appearance and argued that “if humans were not biased to judge others on their appearance, they would not need to remind their children not to judge books by their covers” (p. 408). While frequently warned against ‘judging a book by its cover’, the field of face perception is filled with evidence that suggests that the face does contain a substantial amount of information for evaluators to infer traits. For instance, Kramer and Ward [2] found that four of the Big Five personality traits, as well as physical health, were perceived with some limited accuracy from internal facial features alone and three of the Big Five traits were accurately perceived (just above chance) from just one side of the face. Similarly, Penton-Voak, Pound, Little, and Perrett [3] found that there was some limited accuracy in perceptions of extraversion, emotional stability, and openness to experience when presented with images of composite faces (combining the faces of people with the same personality). Also, research by Little, Burt, Penton-Voak and Perrett [4] found that evaluators were differentially attracted to faces depending on personality traits desired in a partner; that is, “if a trait is desired then faces perceived to possess that trait are found more attractive than faces which do not possess that trait” (p. 1107). Such research highlights potential accuracy in face perception and the relationship between limited accuracy in perceived traits and attractiveness.

Indeed, when investigating the accuracy of perceived intelligence [5] and of perceived health [6] in faces it was found that accuracy was improved to a level above chance when controlling for attractiveness bias. The ‘attractiveness halo effect’ in which desired personality traits are ascribed to attractive people over unattractive people [7] seems to influence the use of attractiveness as a cue when attempting to accurately perceive health or intelligence in faces and is in turn, limiting people’s accuracy. The relationship seems to reflect a *suppression effect*, in which the suppressor (perceived attractiveness) is correlated with the other predictor variable (perceived health or intelligence), but is not related to the dependent variable (actual health or intelligence), so when this noise (relationship between attractiveness and perceived health or intelligence) is controlled for the accuracy in perceptions of actual health or intelligence is increased [8–12].

### Accurate Perceptions of Intelligence and Attractiveness Halo

Kleisner, Chvatalova, and Flegr [5] reported accurate perceptions of intelligence in men’s but not women’s faces. It is important to note that a significant relationship between perceived and actual intelligence was only evident after statistically controlling for perceived attractiveness, though perceived attractiveness itself was not found to be a valid cue to actual intelligence. Kleisner et al. [5] argue that one of the reasons accurate estimations of intelligence are demonstrated in men but not women may be due to the stronger effect of the attractiveness halo in perceptions of female intelligence. These findings highlight the pervasive and detrimental influence of attractiveness on accuracy in attributions.

For decades researchers have debated the accuracy in perceived intelligence and whether attractiveness is a valid cue to actual intelligence [1,5,13–15]. A study by Zebrowitz, Hall, Murphy, and Rhodes [16] found that judgments of intelligence from faces were more accurate than chance for images from childhood, puberty, and middle adulthood, but not more accurate than chance in adolescence or late adulthood. Zebrowitz et al. [16] discussed how facial attractiveness might relate to actual intelligence based on various potential paths: (a) biological, with good genes being inherited; (b) environmental, including the impact of nutrition and healthcare; (c) influence of intelligence on grooming and health decisions; (d) and a self-fulfilling prophecy, in which attractive people are expected to be smarter and given greater opportunities to become smarter. A later study by Zebrowitz and Rhodes [17] investigated the relationship between facial attractiveness and actual intelligence in the upper and lower halves of the attractiveness distribution and reported that, consistent with the ‘bad genes hypothesis’, facial attractiveness was a valid cue to actual intelligence only in the lower half of the attractiveness distribution. Consistent with the ‘anomalous face overgeneralization hypothesis’, attractiveness was used (spuriously) as a cue to intelligence across the entire attractiveness distribution [17]. Thus, participants were accurate in judging intelligence based on attractiveness, but only because faces perceived as unattractive were judged as having low intelligence. These findings are consistent with the ‘bad genes’ hypothesis, which implies that faces perceived as very unusual or unattractive may be an indicator of poor genetic fitness.

A more recent study by Mitchem et al. [18] highlights several problems in previous research investigating attractiveness and intelligence, namely publication bias, inconsistencies in definitions of intelligence and attractiveness, research design flaws, and small sample sizes. They conducted research on the largest sample to date, utilizing a twin dataset and independently collected measures of facial attractiveness and general intelligence. They found no support for a relationship between actual intelligence and perceived facial attractiveness.

### Attractiveness and Academic Performance

Research has also investigated the potential relationship between perceived attractiveness and *actual* academic performance, with no clear consensus. Some investigations have showed that students who are perceived as more attractive achieve higher grades and higher scores on standardized achievements tests (e.g. [19–21]). Other studies failed to find any relationship (e.g. [22,23]).

Nonetheless, the relationship between perceived attractiveness and *perceptions* of academic performance is clear. A meta-analysis conducted by Dusek and Joseph [24] scrutinized fourteen studies investigating physical attractiveness and its relation to teacher expectancy. The review concluded that perceived facial attractiveness is significantly correlated with teacher expectations of academic performance and positive personality attributes. For example, a cornerstone study by Clifford and Walster [25] indicated a significant correlation between physical appearance and teacher expectations. A similar study also suggested a positive correlation between teachers’ ratings of attractiveness and expectations of children’s skills [26] showing that teachers judged children rated as more attractive as more social, confident, popular, academically strong, and more likely to become leaders than students who were rated as less attractive.

Another meta-analytic review by Ritts, Patterson, and Tubbs [27] found that students perceived as attractive are more likely than students perceived as unattractive to be ascribed positive educational traits. Specifically, students perceived as attractive were judged as more intelligent, having more academic potential, and having better grades. It was also noted that other variables such as gender, race, and knowledge of past performance also influenced expectations, but were not significant moderators to the attractiveness influence [27]. Consequently, while there is little consensus and weak supporting evidence for a relationship between perceived attractiveness and *actual* intelligence or academic performance, there is convincing research documenting the relationship between perceived attractiveness and *perceived* intelligence or academic performance.

### Accuracy in Face Perception

Research suggests extroversion can be accurately perceived after only a 50-ms exposure to a face [28], strength can be accurately estimated from faces independent of height, weight, and age [29] and the dark triad of personality (Machiavellianism, narcissism, and psychopathy) can be accurately perceived in composites of expression-neutral facial images [30]. Note here that accuracy does not imply a large effect size; accuracy may be significant, but with performance only slightly above chance. Nonetheless, this limited accuracy is still somewhat impressive given the lack of conventional information (i.e. information about behaviour) that we typically think affects such judgements; thus, the effects may be small but they are still noteworthy. Todorov, Olivola, Dotsch, and Mende-Siedlecki [31] suggests that little time is needed to arrive at a consensus on social attributions from faces, however many studies overstate the validity of these attributions. There are various perspectives on why and how such social attributions from faces are made that explain the potential both for accuracy and for limitations in accuracy.

Biological cues may shed light on how people are rating social judgements at above-chance accuracy from neutral-expression facial images alone. For instance, research suggests the shape of a face is related to the current [32–34] and prenatal [35] levels of testosterone. Research has also suggested that facial adiposity is closely associated with circulating testosterone [36] and that facial adiposity has been shown to be related to perceived health and attractiveness, as well as measures of actual cardiovascular health and proneness to respiratory illness [37]. Further, facial symmetry, and sex typicality in face shape has been shown to be related to disease resistance [38]. Similarly, an average face shape may signal health, as abnormalities that make a face look slightly different from the average may be caused by genetic or environmental stress [17]. Carotenoid coloration in the face has also been found to signify quality of current diet [39]. The face can also provide clues to recent sleep history, with those who are sleep deprived having less eyelid-openness and more downward mouth curvature than those that are well rested [40].

### Health, Attractiveness and Over-generalization

Clearly, the face provides a variety of cues to hormones, health, and sleep status. One thing all of these cues have in common is their relationship to attractiveness. Namely, research investigating attractiveness and the ‘good genes’ theory has argued that facial symmetry [41], averageness [42], sexual typicality [43], eyelid-openness and mouth-curvature [44], carotenoid coloration in the face [39], and adiposity [37] may be attractive because of their relationship to health [45–47].

The link between potential cues to health in the face and perceived attractiveness is one explanation for the ‘attractiveness halo effect’. Research suggests this preference for attractive (or healthy looking) individuals appears early in infancy, with infants as young as two-months old gazing longer at attractive faces over unattractive or unusual looking faces [48,49]. It is unknown whether or not such preferential looking reflects early learning [50–52]. Further, Langlois, Roggman, and Reiser-Danner [53] found that twelve-month-old infants would play longer, have more involvement, experience less distress and withdrawal, and seem to exhibit more pleasure when interacting with attractive people as compared to unattractive people. Also noteworthy is the degree of agreement regarding facial attractiveness. Specifically, studies have shown consistency between men and women regarding opinions of facial attractiveness [45]. Surprisingly, agreement on facial attractiveness is apparent even across different countries [1,54].

In an attempt to investigate whether facial attractiveness provides evidence of actual health, which may partially explain this positive bias towards attractive people, Kalick, Zebrowitz, Langlois and Johnson [6] found that evaluators’ perceptions of attractiveness are actually poor predictors of current or future actual health. While attractive faces were mistakenly rated as healthier than their peers, the correlation between perceived health and actual health increased when attractiveness was statically controlled, implying that attractiveness suppresses the accurate recognition of health.

This improvement in accuracy of health judgments after controlling for attractiveness is similar to the improved accuracy of intelligence judgments when the attractiveness halo is statistically controlled [5]. Indeed there is evidence to suggest a relationship between various health factors and cognitive or intellectual performance. Specifically, it has been found that phobic anxiety [55], trait anxiety [56,57], drug use [58], diabetes [59,60], poor sleep [61], and frailty [62] have been negatively associated with both health and cognitive function in older individuals. Similarly, exposure to chronic aircraft noise [63], infection with parasitic worms [64] and food insufficiency [65] have been found to negatively impact health and cognitive performance in children. Given the close relationship between actual health, actual cognitive performance, and perceived attractiveness, facial cues to health might also be cues to both attractiveness and cognitive ability, leading to correlations between attractiveness and perceived competence. Such correlation might lead to overgeneralization and inaccurate perceptions of academic ability in healthy individuals based spuriously on attractiveness. Hence we explore whether or not the ‘blinded by beauty’ phenomenon found in perceptions of health [6] and intelligence [5] also applies to the perception of academic performance from first impressions of neutral-expression static facial images.

### Theories of Intelligence and Academic Performance

Given the controversy over definitions of intelligence and differences in theories of intelligence [66] it is likely that, in addition to being limited by the attractiveness halo, accurate perceptions of intelligence are also limited by variation in understanding on the meaning of the term ‘intelligence’. While someone who agrees with a fixed theory of intelligence believes there is little a person can do to change their actual intelligence, someone with a growth theory of intelligence argues that intelligence can change over time with the appropriate environment [67–69].

Perceptions of academic performance from faces are likely to suffer similar inconsistences in evaluator perspectives of what factors most influence academic performance. While research has consistently shown that intelligence predicts academic performance [70], it is well documented that the personality trait of conscientiousness is a stronger predictor of academic performance than intelligence [70,71]. Hence, it could be argued that asking evaluators to assess academic performance from faces would yield just as much ambiguity as attributions of intelligence, as consensus would be adversely affected both by disagreement in fixed vs. growth theories of intelligence, and by different perspectives on how much academic performance relies on intelligence versus conscientiousness.

Research on the Intelligence Competence Theory (ICT) further undermines consensus of perceived academic performance by suggesting that people who are less intelligent compensate by becoming more conscientious to reach their goals [72,73]. Thus, some might think a person with a less intelligent looking face is more academically able because the person may work harder to get better grades. Previous studies have highlighted consensus and accuracy of perceptions of most of the big five personality traits from face, yet conscientiousness is sometimes [74], but not always correctly detected [3,75]. Given the relationship between actual conscientiousness and academic performance (compared to intelligence), we explore whether perceptions of conscientiousness are more likely to predict actual academic performance than perceptions of intelligence.

### Research Questions

Research investigating perceptions of academic performance has primarily been concerned with exploring the potential of attractiveness to be a valid predictor of academic performance [19–23,76] and exploring the effects of perceived academic performance on students’ actual performance in the future [24–27]. No research that we are aware of has investigated the potential accuracy of perceptions of actual academic performance from faces when controlling for the attractiveness halo. Given the different perspectives and theories of the term ‘intelligence’ [66,67,77] and the varying perspectives on how much intelligence predicts academic performance compared to conscientiousness [72,73], we hypothesize that evaluators will be more accurate in perceiving actual academic performance when specifically asked to rate conscientiousness than when asked to rate the more ambiguous terms ‘intelligence’ or ‘academic performance’.

Further, it is possible that attractiveness detracts from accuracy in perceptions of academic performance much as attractiveness can detract from accuracy in perceptions of health and intelligence. While there are various seemingly logical explanations for why attractiveness could be a valid cue to academic performance, the empirical evidence for a link between the two is extremely weak and perhaps only existing in the lower half of the distribution (i.e. driven by potential outliers with genetic or developmental problems affecting both appearance and cognitive ability). We hypothesise an ‘attractiveness halo’ in which attractiveness is not linked to *actual* academic performance but is significantly correlated with *perceptions* of academic performance. Further, we hypothesise that controlling for the misperceptions about attractiveness may improve accuracy in perceptions of academic performance.

We argue that this effect of controlling for attractiveness takes the form of a classic type of suppression (see [8–12]). In classical suppression, the suppressor is unrelated to the variable of interest but is related to the predictor, and therefore the shared variance between the predictor (in this case, *perceived* conscientiousness, intelligence or academic performance) and the suppressor (attractiveness) is unrelated to the outcome measure (*actual* academic performance). By controlling for this irrelevant variance in the predictor, the strength of the association between the predictor and outcome variable increases. In other words, controlling for attractiveness may reveal a ‘blinded by beauty’ phenomenon similar to that found in health [6] and intelligence [5] judgments.

## Method

All data collection was approved by UTREC and the School of Psychology and Neuroscience ethics committee (PS1087), University of St Andrews. All participants provided informed written consent and were debriefed accordingly. The individuals in this manuscript have also given written informed consent to blend their facial photographs to create average faces and publish these case details. Written consent was recorded via both electronic submission and on hard copies. The ethics committee approved this consent procedure.

### Facial Stimuli

Students from the University of St Andrews were recruited to take part in an experiment called “Influences in the perception of intelligence in faces” as part of a larger data collection. One-hundred of the most standardized (e.g. clean shaven, neutral expression and head posture) Caucasian faces between the ages of 18 and 24 (*M*_*age*_ = 20.85, *SD* = 2.15; 67 females, 33 males) were chosen as stimuli. The original image collection contained more women than men and removal of males with beards enhanced the gender bias. Nonetheless, we maximised the number of stimuli available for judgments to maintain power in the analysis. Selection of standardized faces was done blind to their academic performance. Todorov and Porter [78] highlight significant differences in person impressions within multiple facial photos of the same person due to random variation and discuss how this can influence accuracy of personality inferences based on faces. Thus, it was important to select the most standardized stimuli. All of the stimuli photographs of participants used were taken under standardized lighting conditions and camera set-up; individuals had their hair pulled back, did not wear any kind of make-up or jewellery, and were instructed to pose with a neutral facial expression. Face images were aligned on left and right pupils. Images were then resized and cropped (1608 x 2584 pixels) so that an equal proportion of hair and neck was exposed in each.

### Academic Performance Measures

All participants consented to releasing their academic performance records for the purpose of this research. Academic records were accessed via the Universities database. Academic performance at the University of St Andrews is marked on a 20-point scale reported to one decimal place for final module grades. An average academic performance was calculated by taking the Grade Point Average (GPA) across every year weighted by every module credit completed by the student. Participants varied in their course of study and the number of modules completed based on their year and semester of study (63 in Sciences, 37 in Arts; 44 first and second year undergraduates, 39 third and fourth year undergraduates, and 17 in postgraduate courses). Accordingly, methods of evaluation (e.g. exam, essay, and dissertation) varied.

### Face Ratings

Four separate groups of participants were recruited and paid via Amazon Mechanical Turk to obtain ratings of perceived attractiveness, intelligence, conscientiousness, and academic performance (no other face ratings obtained for this study). Table 1 shows the demographics of each participant group. Differences in sample sizes were based on differences in the number of participants completing the task while the link was live on Amazon Mechanical Turk and number of exclusions. Participants who reported their ethnicity as different from ‘white Caucasian’ were excluded when calculating the average ratings of perceived attractiveness, intelligence, conscientiousness, and academic performance, as stimuli presented were Caucasian and judgments of other ethnicities may be more susceptible to stereotypes [79]. Analysis was re-run with all participants and there were no differences in the pattern of findings; i.e., all significant results remain significant, and all non-significant results remain non-significant.

**Table 1:** Sample Information.

Participant Group	*M* Age	*SD* Age	Exclusions	Total Sample	Gender
Attractiveness	40.16	12.44	5	32	F = 11 M = 21
Intelligence	40.00	8.99	12	25	F = 16 M = 9
Conscientiousness	42.32	12.17	8	20	F = 10 M = 10
Academic Performance	38.28	12.29	16	47	F = 22 M = 25

Each participant group reflects a separate group of raters for one face perception task.

Female is represented by F and male by M.

Evaluators first previewed all stimuli with each image displayed for one second. The stimuli were then re-presented so that participants could rate the face on the focal trait for each sample: perceived attractiveness, intelligence, consciousness, or academic performance. Faces were presented in random order. To ensure the paid participants were not quickly and hastily clicking through images, images were presented for at least one second before participants were allowed to continue to the next image, but no maximum response time was enforced. Evaluators then completed a questionnaire inquiring about their age, gender, and ethnicity.

Facial ratings were done on a 7-point scale with endpoints according to the face rating task: attractiveness endpoints were *not at all attractive* to *very attractive*; perceived intelligence endpoints were *not at all intelligent* to *very intelligent*; perceived conscientiousness endpoints were *not at all conscientious* to *very conscientious*.; and perceived academic performance endpoints were *very low academic performance* to *very high academic performance*.

Participants who rated perceived academic performance were presented with a statement at the top of each facial image presented asking “Please rate how well you think this person does in University compared to the other people presented”. Participants who rated perceived conscientiousness were presented with a statement at the top of each facial image presented that read “Conscientiousness is the personality trait of being thorough, careful, or vigilant–with the desire to do a task well. Based on the definition of conscientiousness provided–how conscientious do you perceive this face to be compared to the other faces presented”.

## Results

An average score of perceived attractiveness, intelligence, academic performance and conscientiousness was calculated for each of the 100 faces based on the average of all the evaluator ratings. Table 2 gives the zero order correlations between ratings and academic performance and demographic variables. There was a significant correlation between older age and higher actual academic performance and female faces were perceived as more attractive (see Table 2).

**Table 2:** Zero-Order Matrix.

	Actual Academic Performance	1	2	3	4	5
1. Age	.282**	**1**				
2. Sex	-.098	-.011	**1**			
3. Attractiveness	.027	.296**	-.296**	**1**		
4. Intelligence	.072	.302**	-.206*	.807***	**1**	
5. Conscientiousness	.175^†^	.313**	-.360***	.812***	.825***	**1**
6. Academic Performance	.124	.308**	-.150	.738***	.802***	.810***

Zero-order correlations in which rated academic performance is correlated with facial attractiveness, perceived intelligence and perceived conscientiousness. Sex is coded female = 0, male = 1. Correlations are based on 100 faces.

***p < .001.

**p < .01.

*p < .05

^†^*p* < .1.

Two-tailed probabilities.

As predicted, there was no relationship between attractiveness and actual academic performance (*r* = 0.03), but a strong positive correlation between attractiveness and perceived intelligence (*r* = 0.81), attractiveness and perceived academic performance (*r* = 0.74) and attractiveness and perceived conscientiousness (*r* = 0.81).

Given the high correlations between rated attributes (perceived attractiveness, perceived conscientiousness, perceived intelligence and perceived academic performance), we wanted to ensure that any statistical controls were based on sufficiently reliable measures and discriminability valid constructs. Cronbach’s alphas were calculated for perceived attractiveness (32 ratings; *α* = 0.94), intelligence (25 ratings; *α* = 0.86), academic performance (20 ratings; *α* = 0.73), and conscientiousness (47 ratings; *α* = 0.91). After correcting for attenuation due to measurement error [80,81] the relationships between attractiveness and perceived intelligence (*r* = 0.90), between attractiveness and perceived academic performance (*r* = 0.89), and between attractiveness and perceived conscientiousness (*r* = 0.88) were all marginally higher but do not indicate redundancy.

We explored any potential issues with multi-collinearity, as research has suggested high VIF calculations may raise concerns over interpretations [82]. The test to see if the data met the assumption of collinearity indicated that multi-collinearity was not a concern (VIF scores over 10 are seen as problematic; [82]). In this study none of the VIF values were a concern: perceived attractiveness, tolerance = .243, VIF = 4.11; perceived academic performance, tolerance = .236, VIF = 4.24; perceived intelligence, tolerance = .152, VIF = 6.58; perceived consciousness, tolerance = .193 VIF = 5.18).

**Fig 1:**
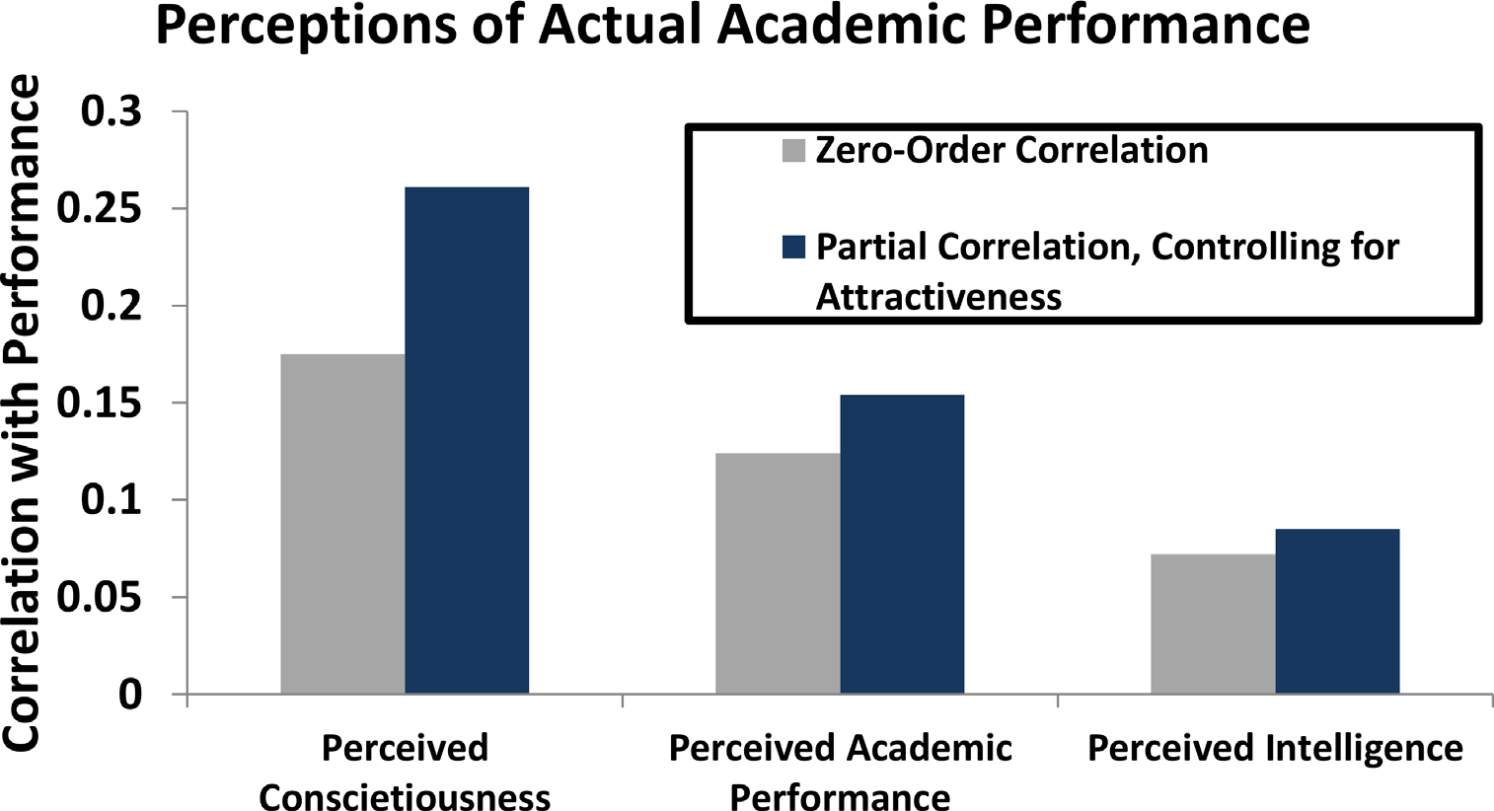
Partial Correlations. This bar graph shows the increased accuracy of the different perceived competence variables when controlling for perceived attractiveness. The same pattern emerges when controlling for the additional variables of sex and age of face.

Partial correlations were conducted in which the influence of age of face, sex of face, and perceived attractiveness were controlled for. Partial correlations revealed (see Fig 1) a significant correlation between perceived conscientiousness and actual academic performance (*r* = 0.22, *p* = 0.035). The partial correlations reveal no relationship between actual academic performance and perceived academic performance (*r* = 0.13, *p* = 0.191) or perceived intelligence (*r* = 0.06, *p* = 0.544). Findings do not change when controlling for only attractiveness in the partial correlation. Nor do they change when controlling for the combination of attractiveness and age or the combination of attractiveness and sex of face.

We investigated the predictive power of perceived conscientiousness over attractiveness and the other perceived competence variables with a multiple linear regression model. In a simple regression model perceived conscientiousness significantly predicted actual academic performance (B = 1.59, SE = 0.712, 95% CI [0.18, 3.00], p = 0.027, β = 0.48), but perceived academic performance (B = -0.03, SE = 0.843, 95% CI [-1.70, 1.64], p = 0.969, β = -0.01), perceived attractiveness (B = -0.89, SE = 0.51, 95% CI [-1.89, 0.11], p = 0.082, β = -0.34), and perceived intelligence (B = -0.07, SE = 0.849, 95% CI [-1.75, 1.62], p = 0.939, β = -0.02) did not significantly predict actual academic performance (overall model: adjusted R^2^ = 0.08, F(4, 106) = 2.13, p = 0.082).

In a multi-step hierarchical model (1st step independent variables: perceived attractiveness, perceived intelligence, perceived academic performance, 2nd step independent variable: perceived conscientiousness) predicting actual academic performance, the second step in the model (perceived conscientiousness) showed a significant increase in variance explained (R^2^ change = 0.45 p = 0.027).

### Facial Averages

**Fig 2:**
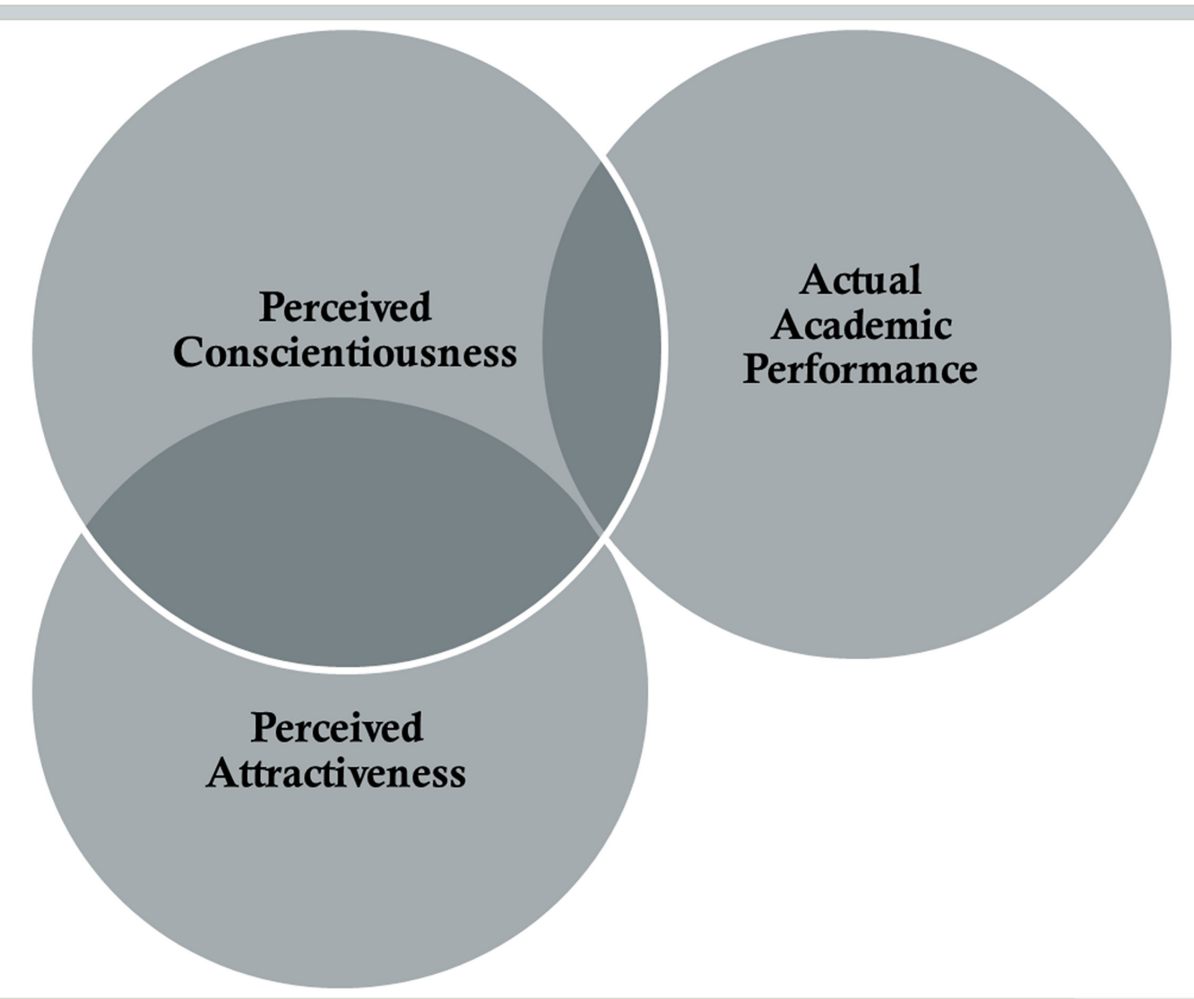
Composite images of percieved consientiousness. The images presented reflect the top and bottom 25% of faces percieved as most (left) and least (right) conscientious. The attractiveness halo would suggest that faces percieved as most conscientious (left) would be more attractive than the faces rated as least conscientious (right).

Facial averages of faces were created to help the reader visualize perceptions of conscientiousness and the attractiveness halo. All face images were manually delineated with 188 points. The averaging (a) computes the average coordinate values for 188 facial landmarks within the set of face images, (b) warps each shape of each facial image into these average coordinates, and then blends the warped component images [83,84]. Facial averages (see Fig 2) were synthesized from the top 25% male and female faces (8 male and 16 female faces) and bottom 25% male and female faces with the highest and lowest scores on perceived conscientiousness [85,86]. These average images were then made symmetrical (see [41]).

## Discussion

There are three main findings. First, there was no first-order relationship between perceptions of conscientiousness, academic performance or intelligence and actual academic performance. Second, when controlling for the expected influences that age, sex and perceived attractiveness on perceptions of competence (perceived conscientiousness, academic performance and intelligence), then the relation between perceived competence and actual academic performance increased in strength. Third, perceived conscientiousness was the single best face perception predictor of actual academic performance (outperforming perceived intelligence and perceived academic performance), and again accuracy was significantly improved when controlling for the suppressor variable of attractiveness.

**Fig 3:**
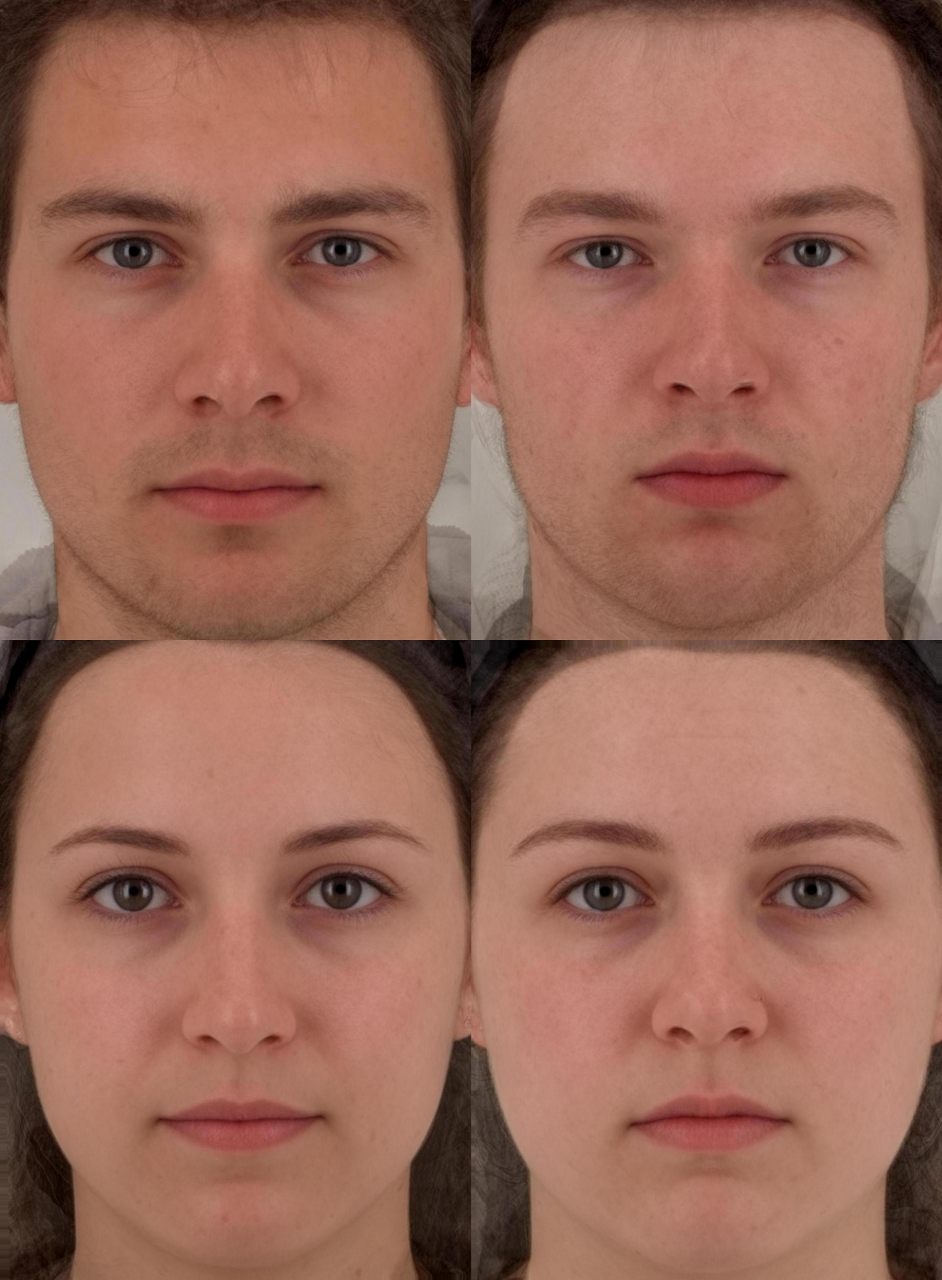
Attractiveness Suppression. This figure shows the noise in perceived conscientiousness (the overlap between perceived attractiveness and perceived conscientiousness) and how by suppressing this noise results in an improved predictor of actual academic performance (greater overlap between the remaining perceived conscientiousness and actual academic performance).

As we expected, the form of the relationship is one of classic suppression in which there is some factor (perceived attractiveness) that is correlated with perceptions of conscientiousness, but not correlated with actual academic performance [8–12]. When this factor is controlled, the relationship between perceived conscientiousness and actual academic performance is increased (see Fig 3). It should also be noted that, although some previous literature suggests weak correlations between attractiveness and cognitive performance measures [16], in our study perceived attractiveness was not a valid cue to actual academic performance. These results suggest that we are ‘blinded by beauty’ in a way in which we would be more accurate in our perceptions of academic performance from faces if we were not influenced by the ‘attractiveness halo’ effect.

Given the amount of research on higher expectations and desired educational traits being ascribed to attractive students over unattractive students, it is not surprising that faces that were rated as more intelligent, having better academic performance and being more conscientious were also rated as more attractive (see composite faces in Fig 2). As predicted, there were high correlations between perceptions of attractiveness and perceptions of intelligence, conscientiousness, and academic performance, likely reflecting the strength of the attractiveness halo, as well as the similarities among these perceived competence measures [87]. While there is less evidence to suggest perceptions of intelligence and academic performance are unique constructs, the possibility that perceived conscientiousness and perceived attractiveness are not distinguishable empirically is dealt with in two ways: face validity of the items for which evaluators were clearly rating conscientiousness or attractiveness (the measures were unambiguous to the evaluators); and we calculated inter-evaluator reliabilities for conscientiousness and attractiveness ratings and even after correcting for attenuation due to measurement error, the correlations between these variables remained distinct (i.e. they were imperfectly correlated). Taken together, these elements suggest that these measures can be treated here as distinct constructs, and that they are measured with sufficient reliability to be distinguished empirically in this study. The high correlations do create potential for interpretative difficulties in multiple regression, and under such circumstances we find it important to emphasize the role of suppression in their relationship in a way that reflects the traditional understanding of the attractiveness halo.

Findings suggest that accuracy in perceptions of academic performance also increases with the clarity and validity of the question proposed. When controlling for attractiveness, age and sex, perceptions of conscientiousness in faces yielded above chance accuracy in predicting academic performance, but accuracy in predicting actual academic performance did not reach levels of statistical significance with perceptions of intelligence or perceptions of academic performance. Given the high correlations between these perceived competence measures, it is difficult to say for certain whether perceptions of conscientiousness are unique in their capacity to predict actual academic performance over and above perceptions of intelligence or academic performance. Rather, it seems perceptions of conscientiousness predicts actual academic performance because, in comparison, it may be the least ambiguous competence construct. As previously argued, it is likely that individual differences in theories and understandings of intelligence can lead, on average, to less accurate perceptions of intelligence in faces. Likewise, perceived academic performance is possibly confounded by a combination of the ambiguities in the term intelligence (fixed vs. malleable) and the limited consensus on how much intelligence (in relation to conscientiousness) is necessary for high academic performance; hence the limited accuracy of perceived academic performance compared to perceived conscientiousness in predicting actual academic performance.

The improved accuracy in perceived conscientiousness predicting actual academic performance over perceived intelligence is also consistent with research that suggests that actual conscientiousness is a stronger predictor of academic performance than actual intelligence [70]. Further, the Intelligence Compensation Theory (ICT) suggests that conscientiousness acts as a coping strategy for relatively less intelligent people. While evidence for ICT is limited, some studies have found significant negative correlations between fluid intelligence and conscientiousness [72,73]. Other studies have found a significant negative correlation between crystalized intelligence and conscientiousness [88]. Thus, our findings of perceived conscientiousness better predicting actual academic performance in faces than perceived intelligence is consistent with literature suggesting actual conscientiousness is a better predictor than intelligence in predicting actual academic performance. Nonetheless, given the high correlations amongst the perceived competence variables explored (perceived intelligence, perceived academic performance and perceived conscientiousness), we must be cautious in claiming that only perceived conscientiousness is related to actual academic performance; rather we argue that the specificity in rating tasks and the influence of attractiveness bias are worth considering when exploring validity of judgements based on faces.

The increased accuracy of academic performance in faces after controlling for attractiveness has important implications. Indeed, Olivola and Todorov [89] showed that judges overweigh aspects of appearance and would be more accurate in judging personality if face perception was ignored. However, facial impressions have consistently been shown to influence our opinions as well as bias decisions in politics [90], leadership [91], law [92], parental expectations and punishments on children [93], military rank promotion [94], and teacher evaluations [95]. Clearly, the power of first impressions is critical and has repeatedly been shown to influence our opinions about a person.

Furthermore, research has found that femininity is considered more attractive than masculinity [43] and that females perform better academically and stay in education longer than males [96], which likely leads to females being ascribed more desired educational traits over men. It is also well documented that older students do better on intelligence tests [97,98] and do better academically than younger students. Moreover, crystalized intelligence and perceptions of wisdom have shown to increase linearly with age [99,100], which would influence impressions of competence in older students (hence the intentionally limited university age range for facial stimuli presented). Our research suggests that when controlling for biases of attractiveness, age and sex, independently or collectively, accuracy of perceived academic performance is significantly improved.

Perhaps one of the most alarming consequences of using insufficient information to guide first impressions is the expectancy effect in education. The classic Pygmalion study conducted by Rosenthal and Jacobson [101] suggests that expectations alone are capable of influencing the targets’ actual performance. Specifically, the Pygmalion study found that students who were arbitrarily assigned the label ‘bloomers’ (i.e., anticipated to show future promise) eventually scored higher on future tests than other students, even though the students labelled as ‘bloomers’ were a random sample and not any more intelligent than the other students in the class. More recent research on expectancy effects by Sorhagen [102] found that teachers’ inaccurate expectations of students in first-grade was associated with students’ academic performance in high-school and that students from lower income families were especially influenced by this bias. Likewise, De Boer, Bosker, and Van Der Werf [103] defined expectation bias as the difference between observed and predicted teacher expectation and found a significant relationship between teacher’s expectation bias of students’ performance and actual performance 5 years later. Hence, perceptions of conscientiousness, intelligence and academic performance may play a vital role in the classroom environment and in the success of a child’s education.

Future research in face perception can benefit from noting the significant differences in perception accuracy based on different theories of intelligence or competence. Perhaps more importantly, given the well documented effects of expectations of academic performance on actual academic performance, our findings help emphasize the biased effects of perceived attractiveness on expectations of academic performance. While it seems unlikely that another person’s attractiveness can be filtered out when attempting to accurately perceive academic performance, the mere knowledge of the negative influence attractiveness has on accuracy may encourage less biased practice; for perhaps the best antidote to deter unconscious bias is to make conscious the possibility of bias.
